# Crucial aspects promoting meaning and purpose in life: perceptions of nursing home residents

**DOI:** 10.1186/s12877-017-0650-x

**Published:** 2017-10-30

**Authors:** Jorunn Drageset, Gørill Haugan, Oscar Tranvåg

**Affiliations:** 1 Department of Nursing, Faculty of Health and Social Sciences, Western Norway University of Applied Sciences, Inndalsveien 28, N-5005 Bergen, Norway; 2 Centre for Elderly and Nursing Home Medicine, Department of Global Public Health and Primary Care, University of Bergen, Bergen, Norway; 3 Department of Public Health and Nursing, Norwegian University of Science and Technology, Trondheim, Norway; 4 Center for Health Promotion Research, Norwegian University of Science and Technology, Trondheim, Norway; 5 Norwegian National Advisory Unit on Women’s Health, Oslo University Hospital, Rikshospitalet, Oslo, Norway

**Keywords:** Meaning, Purpose in life, Nursing home residents, Well-being, Belonging, Treasured activities, Spiritual connectedness, Nursing, Quality of life

## Abstract

**Background:**

Meaning and purpose in life are fundamental to human beings. In changing times, with an aging population and increased life expectancy, the need for health care services and long-term care is likely to grow. More deeply understanding how older long-term care residents perceive meaning and purpose in life is critical for improving the quality of care and the residents’ quality of life. The purpose of this study was to explore crucial aspects promoting nursing home residents’ experience of meaning and purpose in everyday life.

**Method:**

An exploratory hermeneutical design with qualitative interviews for collecting data.

**Results:**

Four key experiences were found to promote meaning and purpose in life: 1) physical and mental well-being, 2) belonging and recognition, 3) personally treasured activities and 4) spiritual closeness and connectedness.

**Conclusion:**

In supporting meaning and purpose in life of nursing home residents, the residents’ everyday well-being should be a central focus of care and facilitate personally treasured activities. Focused attention should also be given to the meaning-making power of experiencing belonging, recognition and spiritual connectedness.

## Background

Life expectancy is increasing worldwide [1], and the aging population is likely to increase the need for health care services and long-term care. The proportion of people 67 years and older living in institutions in Norway is 5.1% and 69.6% for people older than 80 years [2]. The health status of people living in nursing homes is often complex. Chronic illnesses and multiple diagnoses are common [3, 4], involving somatic diseases and/or mental disorders. The nursing home population has high age, 2 years as the mean duration of stay and a high mortality rate. Among nursing home residents, 80% have dementia [3]. Further, the residents may also be exposed to stressful events such as loss of home and losing relational closeness to their spouse, relatives and friends. Any or all of these conditions may influence the residents’ well-being and quality of life.

Meaning is crucial for overall satisfaction among nursing home residents [5] and for spiritual, functional and social well-being [6]. Purpose in life as a concept originates from Frankl’s work, perceiving meaning as a motivating and vitalizing force in human life [7]. Finding meaning is feeling that life is of significance, worthwhile or targeted [7]. Purpose in life refers to intention: an aim to be met or to be achieved, and meaning refers to making sense of or establishing coherence in one’s existence [6, 8]. Those who fail to find purpose in life may experience total meaninglessness, a state that Frankl [7] termed an existential vacuum. According to Frankl [7], meaning and purpose are used synonymously. Meaning and purpose in life represent a set of attitudes and views that make the world intelligible, such as having goals to strive for by working creatively, performing work or a task that is experienced to be meaningful, finding solutions in difficult life situations, having someone to love, experiencing nature and culture and enduring suffering. In the context of suffering, meaningful implies people’s inherent ability to turn a personal tragedy into human growth and to transform despair into well-being by means of one’s chosen attitude. Being human is about shaping and reshaping oneself [7]. In this light, the phenomenon of self-transcendence is clearly closely related to purpose and meaning in life [7].

Self-transcendence is the ability to transcend oneself by opening one’s mind to something outside oneself [9, 10]; connectedness (belonging) is the core of self-transcendence [10]. Hence, caring about someone or something outside oneself results from the ability to transcend oneself and provides meaning. Self-transcendence is a strategy to distance oneself and one’s situation and thus signifies distancing oneself from one’s problems and ailments. This distance enables one to see and experience the situation from a different perspective and thus an opportunity to find meaning amid losses and painful or difficult circumstances [9].

Nursing literature [9–11] commonly addresses both self-transcendence and meaning in life, underpinning the importance of nurses helping people and their families not only in coping with illness and suffering but also finding meaning in these experiences [9–12]. However, finding meaning in life is a subjective and unique process within the individual [9]. Meaning cannot be invented; the individual must perceive it. A nurse cannot tell or show people how they can find meaning. Nor can nurses create, point out or transfer meaning to another person; finding meaning in life requires an inner process and active participation. Nevertheless, nurses can walk along with people, supporting the meaning-making process by empathic listening and questioning. According to Starck [9] based on Frankl [7], one way to experience meaning is to open up to other people’s care, kindness and love. Positive emotions and moods have been seen to be a stronger source of meaning than activities towards realizing certain goals [13]. Nursing care and nurse–patient interaction therefore have the potential to act as meaning-promoting resources by facilitating positive emotions and moods.

The experience of meaning is fundamental to humans [14]. Meaning and purpose in life among nursing home residents without dementia are significantly related to nurse–patient interaction or to family and friends outside the nursing home or other residents [15, 16]. Moreover, meaning is also significantly related to quality of life, and the nurse–patient interaction represents an essential resource promoting nursing home residents’ physical and emotional health and global well-being [6, 16, 17]. Meaning and purpose in life are parts of intrapersonal self-transcendence, which is fundamental to nursing home residents’ well-being and quality of life [17, 18], are crucial to a positive view of life, self-esteem and happiness and are a crucial aspect of religiosity and spirituality [19]. Nursing home residents have identified connectedness as a core factor that contributes to meaning in life [20]. Meaning is therefore derived through connectedness – inwardly and outwardly – via relationships by communicating with other people, self-reflection on responsibilities, inner dialogue and completing unfinished business [21–23]. The experience of meaning in everyday life in a nursing home is related to residents’ physical and cognitive capability, a sense of belonging and feeling needed [20, 21]. Meaningful communication is an important meaning-making activity for nursing home residents. In accordance with this finding, nursing home residents’ lack of self-determination harms their meaning in life [24]. Respect for autonomy and self-determination is a central principle in nursing ethics and is strongly connected to quality of life and sense of value [25]. Further, autonomy and self-determination are important qualitative indicators of how older people perceive their lives in an institutionalized facility. Niu et al. [26] found that the quality of relationships with family and friends and perceived health are important for experiencing meaning among older institutionalized veterans in Taiwan.

Nursing home residents’ experience of meaning and purpose in life needs to be more deeply understood to improve the quality of care and their quality of life. This qualitative study exploring meaning-promoting aspects among nursing home residents is part of a larger mixed-methods project that has previously documented both positive attributes (sense of coherence, social support) and negative attributes (loneliness, loss, symptoms of depression and sadness) [4, 27] among older people with cancer living in nursing homes.

## Objective

The aim of this study was to identify and describe crucial aspects promoting nursing home residents’ experience of meaning and purpose in everyday life. Such insight is critical for improving nursing home residents’ quality of life and the quality of care in nursing homes.

## Methods

### Design

An exploratory design using qualitative interviews is an advantageous approach in exploring underlying aspects of a phenomenon about which knowledge is limited [28, 29]. This approach has previously been shown to be appropriate for collecting data on experiences among frail older people [30, 31], including those with dementia [32, 33]. We used Gadamer’s hermeneutical methods [34, 35] for analyzing and interpreting the data.

### Study participants

This study is part of a study conducted in 2004–2005 [36], with follow-up until 2011. The inclusion criteria were: people 65 years and older; being capable of carrying out a conversation; residing in a nursing home for at least 6 months; and without dementia: a Clinical Dementia Rating (CDR) ≤0.5. CDR [37] was developed as a staging instrument for dementia and is scored as no (0), questionable (0.5) and mild (1), moderate (2) and severe (3) dementia. The overall level of dementia is derived by using a standard algorithm [38]. Exclusion criteria were: people living less than 6 months in a nursing home, CDR score > 0.5 and those who had their general health status assessed by a doctor or nurse indicating that the resident could not converse with the researcher. At the end of the follow-up, 19 nursing home residents were still alive; 18 were included, among whom 11 were women. The participants’ ages ranged between 77 and 92 years. The participants gave informed consent to participate at each interview, which included allowing the researchers to access their medical records.

### Context of data collection

JD interviewed all 18 participants once in the respondent’s room or in other appropriate locations within the nursing home facility. A temporary and modifiable interview guide was used as a structural framework. The main questions guiding the conversations were: What brings meaning into your life? When do you experience meaning in your everyday living? Based on your perception, can personal meaning be weakened or lost? Moreover, follow-up questions added depth and richness to the previously collected data: Can you tell me more about how this aspect affects you? Can you tell me about a situation in which you experienced this? Are other aspects in this important and meaningful to you? All interviews were recorded on an MP3 recorder and transcribed verbatim.

### Data analysis and interpretation

We analyzed and interpreted the data according to Gadamer’s [35] hermeneutical approach. As highlighted by Fleming et al. [34], researchers conducting studies anchored in Gadamer’s hermeneutical methods must identify and reflect on their own preconceptions of the topic being investigated. This is required for researchers to move beyond their preconceptions and to develop new and deeper understanding of the phenomenon investigated. In this study, all researchers were experienced in nursing care for nursing home residents as well as conducting research and teaching nursing students about older people’s experiences and perceptions. Our preconceptions were grounded on the perception of meaning and purpose in life as a basic human need. We also shared the preconception that the aspects promoting meaning and purpose in the everyday life of nursing home residents are individual and specific to each person. Moreover, we also shared the preconception that meaning-promoting aspects may have universal characteristics: that is, typical features or traits common for most people.

Following Gadamer’s hermeneutical methods [35], we initiated our process of data analysis and interpretation by reading each interview text to get an initial overview of each text and the texts as a whole. Then we read each text several times while noting phrases and keywords during each reading. In this phase, our preliminary analysis indicated that meaning and purpose in life could be related to “experiencing mental and bodily comfort – feeling safeguarded and cared for by the nursing home staff”, “experiencing psychosocial confirmation”, “having the opportunity of taking part in meaningful activities” and “having a spiritual meaning-promoting source”. We identified and explored the essence of each account and developed themes and subthemes on this basis. Exploring each new interview text made those already read more understandable while adding new perspectives to our understanding. Lastly, we explored the transcribed interview text as a whole to search for increased understanding of the patterns and the meaning of the data material as a whole not identified in the individual interview texts.

Hermeneutical methods [35] also involve theoretical interpretation of the collected data. Reading theoretical works concerning meaning, such as the existential analysis of psychiatrist Viktor Frankl [7] and the nursing theories of Starck [9] and Reed [10], helped increase the theoretical understanding of the data material and were chosen as a framework for our theoretical interpretation and discussion.

## Results

Four main themes were crucial for the study participants to experience meaning and purpose in their everyday lives in the nursing home: 1) experiencing physical and mental well-being, 2) experiencing belonging and recognition, 3) experiencing personally treasured activities and 4) experiencing spiritual connectedness and closeness.

### Experiencing physical and mental well-being

This study shows that the experience of well-being, physically as well as mentally, was related to the nursing home residents’ sense of meaning and purpose in everyday life. Maintaining personal hygiene through the gentle assistance of care personnel, having one’s personal preferences and needs met and nurturing hope for regaining or improving health were crucial meaning-promoting aspects.

#### Maintaining personal hygiene through the gentle assistance of care personnel

Many participants required assistance to get out of bed, wash and dress each morning. The nursing home residents experienced the gentle nursing assistance as a meaningful way to start their day and an opportunity for maintaining personal hygiene and well-being. If their individual needs were cared for in an attentive and sensitive manner, the participants experienced the cooperation with the health care workers as meaningful:

They come and help me. Care for my needs throughout the day … at night too [laughs] … it’s very nice … they take good care of me … so I can get up each morning …

#### Having one’s personal wishes and needs met

The experience of well-being was also anchored in personal wishes and needs. Accordingly, experiencing well-being and meaning in daily living was related to physical, interpersonal and intrapersonal factors experienced by the individual:

Of course, I need enough sleep, to eat properly … talk with my family … and try to avoid ugly or unpleasant thoughts.

#### Nurturing hope for regaining or improved health

Participants’ experience of life as meaningful was also seemed connected to their hope of improving their health – restoring their physical well-being and the ability to care for themselves in the future:

Yes, meaning … yes, and I hope that my leg will get better.

… so I can dress and care for myself … it’s very meaningful to me, you know, so I can learn to be more self-reliant.

A positive outlook provided an opportunity to view current health issues and challenges as temporary, a meaningful catalyst of hope for better days to come.

### Experiencing belonging and recognition

The results indicate that belonging and feeling recognized by others were connected to the nursing home residents’ experience of meaning and purpose in life. Feeling part of a communion with family and friends and having a confirming fellowship with the personnel were identified as crucial meaning-promoting aspects.

#### Feeling part of a communion with family and friends

Contact with family was vitally important to the participants; continuing close family ties – belonging – was crucial and an essential meaning-promoting aspect. Opportunity to reminisce together with family, or share time with old friends, contributed to meaningful experiences in daily living, providing opportunities to reconnect with past acquaintances – people who had been and still were important in their lives:

… it’s so very important – having contact with my family … brightens up my whole day … you become another person because you’re able to chat about things that interest you … it makes your world come alive again.

Being together with old friends and family … makes life meaningful.

#### Having a confirming fellowship with the personnel

Living in a nursing home, most of the participants’ daily interpersonal contact was with health care personnel. Their daily caregivers thus became very important people in their lives. Experiencing good relationships with health care workers, discernable by friendliness and mutual respect, contributed towards positive and uplifting fellowship, promoting meaning and purpose in everyday life.

… friendship between health care workers and residents … has been very good … absolutely. Friendship – being on the same frequency with your caregiver – the feeling that he or she respects you, recognizes your self-worth, and likes you as a person … this is true kindness ….

… when they say kind things about you, adjust the pillows and ask if you are lying okay … are polite … and say “good morning” and “good night” ….

The experiences that health care workers shared their compassion and charity by their attitudes and actions confirmed the nursing home residents as esteemed human beings.

### Experiencing personally treasured activities

This study shows that experiences of daily activities that were treasured by the individual nursing home residents strengthened their personal sense of meaning and purpose in life. Enjoying outdoor life, sharing valuable togetherness and generating valuable aloneness were crucial meaning-promoting resources.

#### Enjoying outdoor life

For nursing home residents, much of the daily routine took place indoors within the facility. For participants, opportunities to go outside for a walk in the fresh air were important and a treasured activity:

Leaving the building and walking outside together with others … is crucial ….

#### Sharing valuable togetherness

In their everyday life, the nursing home residents experienced opportunities for socializing and friendly interactions when suitable environments were provided. Participating in stimulating gatherings and enjoyable activities encouraged the nursing home residents’ experiences of meaningful togetherness with others.

… the fact that [the health care workers] arrange a variety of activities you can attend and meet other people … is very positive, with music and exercise for example … and to be able to join in ….

#### Generating valuable aloneness

In this study, several participants said that they were spending much time on their own, often doing nothing. Having the opportunity to participate in personally treasured activities – also when alone – was therefore a crucial foundation for generating meaningful experience in everyday life.

… listening to the radio. I cannot read any more … nor can I write … but I often listen to music … and enjoy the radio ….

When the residents were on their own, opportunities to receive impulses from outside the nursing home facility were beneficial for finding meaning in their everyday lives.

### Experiencing spiritual connectedness and closeness

The results indicate that, for some nursing home residents, a sense of spiritual connectedness and closeness to their god helped in sheltering and strengthening a personal sense of meaning and purpose in life. Having a religious conviction and feeling the mercy of one’s god were essential meaning-promoting aspects.

#### Having a religious conviction

Some participants explained how their religious conviction helped them feel connected to a higher power beyond the material world. Trust in God contributed to meaning in their lives amid illness and suffering.

One’s attitude in all areas of life helps make a difference. Religion for example … that there is … that one has something more ….

#### Feeling the mercy of one’s god

Some found meaning in their experience of closeness to God, through faith in God’s purpose of their lives – also when faced with illness and suffering. Nursing home residents’ transcendental experience of spiritual closeness to God alleviated suffering:

I pray to God that he will help me … and he helps me, he never says no … I only need to wait and see, for God has surely a divine purpose for me and the world.

Experience of meaning in suffering was fostered through faith as well as by turning to God, asking for help – representing a transcendental experience of being listened to, taken seriously, cared for and loved, solely because of being human, a child of God.

## Discussion

This study explored crucial aspects promoting meaning and purpose in life among nursing home residents in Norway. By doing so, this study enabled deeper exploration of the phenomena of quality of life [5, 8, 17, 39] and health in a nursing home population. By exploring meaning-making resources among nursing home residents, this study contributes to a holistic and salutogenic nursing perspective that aims to support well-being, quality of life and health among nursing home residents. This study expands on previous studies by providing empirical knowledge, showing that nursing home residents’ perceived meaning relates to well-being, belonging, being acknowledged and valued by personnel and family, spiritual connectedness and participation in personally treasured activities; these activities give meaning to the individual.

The results reveal four main meaning-making resources, involving nursing home residents’ experiences of: 1) well-being, 2) belonging and recognition, 3) personally treasured activities and 4) spiritual closeness and connectedness to one’s god.

### Well-being

The relationships between meaning and physical, emotional, social, functional [6, 16] and spiritual well-being have been reported among nursing home residents. Moreover, meaning is a significant aspect of both physical and mental well-being for many older adults in general, including those living in nursing homes [6, 8, 40]. The present results signify that the experience of well-being is related to personal hygiene and having one’s personal preferences and needs met, in a context of gentle nursing care. In accordance with Starck’s nursing theory [9], this finding indicates that the positive state of well-being – related to meaning – is achieved by nursing the body as well as the tenderness and awareness being expressed as part of the nurse–patient interaction. The qualities embedded in the nursing care – both regarding nurses’ clinical responsibilities and the nurses’ way of being present – are important to nursing home residents’ well-being [16] and therefore meaning [9]. The present study also disclosed a link between well-being and the nurturance of patients’ hope for an improved state of health and are in accordance with previous studies showing significant associations between meaning, hope and well-being among older people in nursing homes [17].

### Belonging and recognition

Belonging and recognition, related to communion with family, friends and the nursing home personnel, represent the second meaning-making dimension found. In accordance with Frankl [7], meaning and purpose in life involve having someone to love and care about. For most people, these are our family and friends. This finding of belonging and recognition is supported by previous studies demonstrating that relationships with family and friends are fundamental to meaning and the quality of life among nursing home residents [20, 41, 42]. Further, previous research has revealed the significance of how nursing home residents perceive nurse–patient interaction, revealing significant influence on their experience of hope [18], meaning in life [16] and self-transcendence [43], all of which significantly affect well-being.

Nursing theory [9] emphasizes the importance of nurses in helping patients and their families to find meaning amid experiences of illness, losses and suffering. Nursing is always part of a nurse–patient relationship [9, 10], and the nurses can empower or disempower patients. The more vulnerable and dependent on care the nursing home residents are, the more the nurse–patient interaction affects residents’ self-transcendence, quality of life and thus well-being [10]. Previous research has revealed that nursing home residents stress their need for connectedness to the nursing personnel; their definition of close relationships with the nursing home caregivers was based on nurses’ attitudes and behavior [44, 45]. Frankl [7] and Starck [9] state that the experience of other people’s care, kindness and love promotes meaning. Correspondingly, qualitative good nursing care promotes positive states of meaning and mental and emotional as well as physical well-being [6, 9]. Meaninglessness results from violating individuals’ sense of worthiness in the nurse–patient interactions. Thus, the nature of the nurse–patient interaction in nursing homes is critical. Care providers have to be aware that residents interpret their attitude, appearance and behavior as confirming the residents’ worthiness or worthlessness [20, 46].

### Personally treasured activities

The third meaning-making dimension included enjoying outdoor life, sharing valuable togetherness and valuable aloneness. In accordance with previous research, as well as nursing theories on human’s basic needs, outdoor life, fresh air and some physical activity are important to people’s well-being, health and quality of life. However, to the authors’ knowledge, studies on the outdoor activities of frail nursing home residents as well as activities in general are scarce [43, 47]. Studies on nursing home activities in relation to the quality of life have focused on residents with dementia, and the possibility for them to enjoy outdoor life substantially and positively affects their quality of life [48, 49].

Frankl [7] considered meaning and purpose in life to be related to working creatively, performing tasks that are experienced to be meaningful and experiencing nature and culture. Individualizing the daily life experiences of nursing home residents requires offering meaningful activities that reflect their preferences and encouraging them to explore and choose from among these options. Many individuals transitioning to long-term care, however, experience a narrowing of choices for meaningful activities, especially in facilities in which health care workers lack information about residents’ preferences [16, 47, 50]. Residents’ quality of life can be improved if health care workers can discern residents’ preferences and offer them a selection of personally meaningful activities [16, 50]. Consequently, the care personnel need knowledge about the residents’ personal preferences: that is, they need to be involved in meaningful dialogues with the individual nursing home residents and their families. Such dialogues, focusing on what is meaningful to each resident, are meaning-making [9]. Care professionals should help residents to engage in sound communion and personally treasured activities through meaningful dialogue, either with their own friends, family or companions in the ward.

Similar to most people, nursing home residents also need to spend time in silence, alone, speaking with themselves. Accordingly, the present results highlight the importance of valuable togetherness as well as valuable aloneness.

### Spiritual connectedness and closeness

The fourth dimension – spiritual connectedness and closeness – implied having a religious conviction as well as the experience of God’s mercy in one’s life. The faith and inner conviction that a higher being such as God is caring for one’s life and personal well-being represents an inner state of confidence, hope, certainty and shelter from which a state of inner peace and meaning is derived [51]. Such positive emotions as mentioned above are related to inner strength [52, 53] and a positive outlook in life, both of which promote health and coping. The salutogenic theory of what creates health [54] states that meaning is a vital aspect of the salutogenic concept of sense of coherence. Sense of coherence represents a positive outlook in life, comprising that one’s life situation is perceived to be understandable, comprehensible and meaningful. Thus, having a powerful God on one’s side in difficult times and while facing death might bolster an individual’s experience of meaning, sense of coherence and therefore also coping and well-being [55, 56].

Living in a nursing home probably signifies challenges in accepting and adapting to one’s life situation, with the intention of realizing meaning and well-being. In accordance with Frankl [7], finding solutions in difficult life situations and enduring suffering are considered meaning-making resources. Given Frankl’s writings, the will to achieve meaning might embody a valuable attitude or even a goal to strive for in this life situation. From a nursing theory perspective [9, 10], meaning represents a view that makes the world intelligible and denotes people’s inherent ability to turn a personal tragedy into human growth, transcending despair and suffering to reach hope, meaning, self-transcendence and thus well-being. As one informant said: “I pray to God that he will help me … and he helps me, he never says no.” Knowing that a powerful God is listening to one’s prayers in the manner of a resiliently caring, empathic and loving Father might strongly embody a source of social support, which has shown to significantly affect nursing home residents’ quality of life and coping [54]. Further, this informant emphasized that “… for God has surely a divine purpose for me and the world.” This statement illustrates an inner state of meaning and purpose in life derived from one’s personal faith in God. Accordingly, such a positive relationship with and expectations of God exemplify a health-promoting resource among nursing home residents.

### Methodological considerations and study limitations

Qualitative interviews proved suitable for rich data collection based on fruitful dialogue with the study participants. The interviews were audiotaped, ensuring verbatim interview transcription. We explored the transcribed interview texts separately, in relation to each other and as a whole. Through dialogue with the texts, the interpretive process opened up deeper insights into the participants’ perceptions. Although disconfirming evidence was sought throughout the interpretive process, the hermeneutical circle movement [35] helped us move beyond our preconceptions towards new and deeper understanding of the nursing home residents’ experience of meaning and purpose in life.

The study has limitations. Conducting only one interview with each participant is a limitation. The sample was limited, and the results cannot be generalized to the entire population meeting the inclusion criteria. Thus, crucial qualities enhancing meaning and purpose in life probably exist within the total population not documented in this study.

## Conclusion

Nursing home residents have experienced many losses and illnesses. They are frail and face death. Finding meaning and purpose in life in this situation can prove difficult; nevertheless, evidence has shown that individuals who find meaning despite losses, suffering and illness withstand their plagues and the various outcomes better than those with a low sense of meaning.

In a holistic perspective, this study provides deeper insights into the phenomenon of meaning and purpose in life among nursing home residents in Norway. Based on 18 qualitative interviews with 18 nursing home residents, this study explored experiences of meaning and purpose in everyday life in nursing homes. Four key meaning-making experiences were discovered: well-being, belonging and recognition personally treasured activities and spiritual closeness and connectedness to one’s god. Consequently, the well-being of the residents should be a central focus of nursing home care as well as facilitating meaningful activities, spiritual closeness and patients’ experience of belonging and being recognized as a unique person. Correspondingly, nursing home care includes not only care for the body but also for the person inside the body. Meaning and well-being result from positive emotions derived from competent nursing care, empathetic nurse–patient interaction and the experience of connectedness and belonging to other people (family, friends, companion in the nursing home) and God. Nursing home care personnel need support in advancing their understanding of meaning in life as a vital phenomenon, essential to well-being and health in this vulnerable population. Further, nursing home professionals’ skills in empathetic nurse–patient interaction should be supported and facilitated as well as their awareness of what activities – alone or together with others, inside or outdoors – provide meaning for the individual nursing home resident. Providing personal treasured activities in nursing homes should be given priority.
